# Atg27p co-fractionates with clathrin-coated vesicles in budding yeast

**DOI:** 10.17912/micropub.biology.000380

**Published:** 2021-03-29

**Authors:** Verónica A. Segarra, Anupam Sharma, Sandra K. Lemmon

**Affiliations:** 1 Department of Biology, High Point University, High Point, NC, USA 27268; 2 Department of Microbiology, University of Georgia, Athens, GA, USA 30602; 3 Department of Molecular and Cellular Pharmacology, University of Miami Miller School of Medicine, Miami, FL, USA 33101

## Abstract

Atg27p, a single-pass transmembrane protein that functions in autophagy, localizes to a variety of cellular compartments including the pre-autophagosomal structure, late Golgi, vacuolar membrane, as well as early and late endosomes. Its cytoplasmic C-terminus contains a tyrosine sorting motif that allows for its transport to the vacuolar membrane and an additional sequence that allows for its retrieval from the vacuolar membrane to the endosome. Since clathrin is well known to mediate vesicular transport in the endomembrane system, the trafficking of Atg27p and its tyrosine sorting motif suggested that it might be trafficked inside clathrin-coated vesicles (CCVs). In our previous studies, Atg27p was identified by mass spectrometry as a potential component in CCVs, as it was present in CCVs isolated from both WT and auxilin-depleted cells. We now confirm that Atg27p is a component of CCVs using immunoblotting and additional mass spectrometry data.

**Figure 1. Atg27p co-fractionates with clathrin-coated vesicles in budding yeast, while Atg9-containing vesicles are smaller and elute in a later peak:**
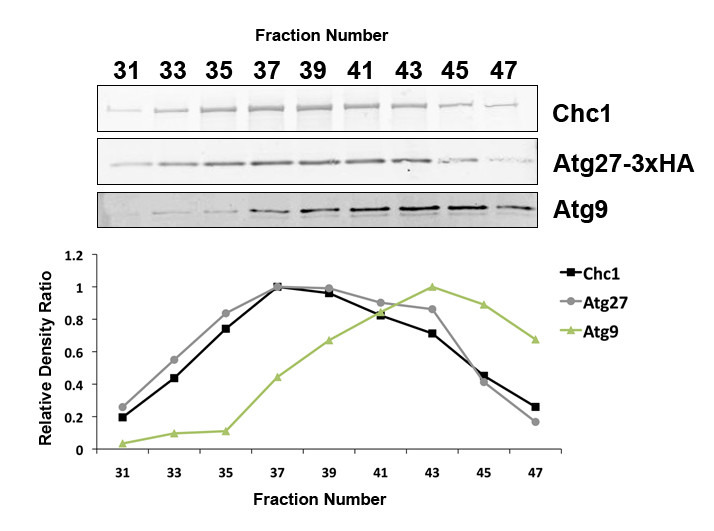
**C**lathrin-**c**oated **v**esicle (CCV)-enriched fractions were harvested from budding yeast (SL5893) and immunoblotted with antibodies to clathrin heavy chain (Chc1p), **h**em**a**gglutinin (Atg27p-HA), and Atg9p for detection of clathrin, Atg27p, and Atg9p, respectively. Densitometry (Image J) was used to calculate relative intensity of each Chc1p, HA, and Atg9p band. The relative intensity of each band was then divided by that of the strongest band (present in fraction #37 for both clathrin and Atg27p; fraction #43 for Atg9p) in each set to generate the *Relative Density Ratio* and plotted on the graph shown.

## Description

Atg27p is a single-pass transmembrane protein that functions in the process of cellular self-eating or autophagy (Yen *et al.* 2007, Segarra *et al.* 2015). Atg27p localizes to a variety of cellular compartments including the pre-autophagosomal structure (PAS), late Golgi, vacuolar membrane, as well as early and late endosomes (Segarra *et al.* 2015). In fact, its short cytoplasmic C-terminus has been found to contain a tyrosine sorting motif that allows for its anterograde transport to the vacuolar membrane and an additional 15-residue sequence that allows for its retrieval or retrograde transport from the vacuolar membrane to the endosome (Segarra *et al.* 2015, Suzuki and Emr 2018). Since clathrin is well known to mediate vesicular transport in the endomembrane system, the trafficking itinerary of Atg27p and its tyrosine sorting motif suggested that it might be trafficked inside clathrin-coated vesicles (CCVs).

In our prior studies, cellular fractions enriched for clathrin-coated vesicles (CCVs) were isolated from budding yeast (Lemmon *et al.* 1988; Ding, Segarra *et al.* 2016). Clathrin-containing samples from the leading half of the clathrin peak were subjected to mass spectrometry analysis to identify new CCV adaptors and cargo (Ding, Segarra *et al.* 2016). Atg27p was identified as a potential component in CCVs, as it was present in CCVs isolated from both WT and auxilin-depleted cells (Ding, Segarra *et al.* 2016). This micropublication further confirms Atg27p is a component of CCVs. First, Atg27p was absent from analogous fractions isolated from yeast cells deleted for the clathrin heavy chain gene (see proteomic analysis in Extended Data files 1 and 2). Second, immunoblotting confirmed that Atg27p co-fractionates with CCVs (Figure 1) but is absent from comparable fractions in *chc1∆* cells. We also found that a separate population of smaller vesicles containing Atg9p elutes from the column slightly later than CCVs (Figure 1).

Fractionation experiments have both clathrin heavy chain and Atg27p peaking together in fraction #37 (Figure 1), strongly suggesting that Atg27p is a novel CCV component. This elution behavior is characteristic of clathrin and CCV components (Lemmon *et al.* 1988). Also, the presence of Atg27p in CCVs is consistent with its reported localization and trafficking itinerary as described above. Moreover, Atg27p has been shown to undergo retrograde trafficking from the vacuolar membrane-to-endosome (Suzuki and Emr 2019) and from the endosome-to-Golgi (Ma *et al.* 2016, Suzuki and Emr 2019) in a SNX-BAR-dependent fashion. Atg27p transport in CCVs (Figure 1) suggests that some of these retrograde transport steps might be clathrin-dependent, as clathrin is well known to mediate vesicular transport in the endomembrane system. Further experiments are needed to ascertain whether or not this is the case (Seaman 2019). Interestingly, Atg27p has also been shown to be an AP-3 cargo (Segarra *et al.* 2015, Suzuki and Emr 2019), linking it to a pathway that is generally considered to be clathrin-independent in budding yeast (Cowles *et al.* 1997, Stepp *et al.* 1997).

A slightly smaller, Atg9p-containing vesicle species elutes from the size-exclusion column and peaks in fraction #43 (Figure 1). These vesicles are likely clathrin-independent Atg9p vesicles. CCVs can range in diameter from 60-200 nm (Pearse and Crowther, 1987) while Atg9p vesicles have a diameter of 30-60 nm (Yamamoto *et al.* 2012). These reported size ranges are consistent with the elution order of these vesicles from the size-exclusion column. Atg9p vesicles have been shown to bud off of the late Golgi, contain Atg27p, and contribute to autophagosome formation at the PAS (Yamamoto *et al.* 2012, Kakuta *et al.* 2012).

Given what is known about the presence of Atg27p in Atg9p vesicles, it is interesting that transport in CCVs seems to be unique to Atg27p (Figure 1). Hence, while Atg27p and Atg9p have trafficking itineraries that seem to intersect or overlap at the late Golgi (Yamamoto *et al.* 2012, Kakuta *et al.* 2012), there are parts of their trafficking itineraries that are distinct.

**Supplemental Materials**

**Extended Data File 1**. CCVs clathrin null strain, all mass spectrometry hits, sorted, and with abbreviated information per slice;https://tinyurl.com/yy6fbv4x

**Extended Data File 2.** CCVs clathrin null strain, all mass spectrometry hits with full information, one slice per tab; https://tinyurl.com/y25z9eum

## Methods

***Yeast***

Standard methods and media were used for genetic manipulations, growth, and transformation of yeast (Guthrie and Fink 1991). *Saccharomyces cerevisiae* strains used in this study are listed in the table below. The Longtine method was used for yeast construction (Longtine *et al.* 1998).

**Yeast strains used in this study**

**Table:** 

**Name**	**Alias**	**Genotype**	**Figure**	**Reference**
SL5893	*aux^–^* Atg27-3xHA	*MAT*α *leu2 ura3-52 trp1 his3-*Δ*200 GAL1:AUX1::TRP1 ATG27-3xHA::HISMX6*	1	This paper
SL12	*chc1∆*	*MATa leu2 ura3-52 trp1 chc1∆::LEU2 scd1-v*	Tables S1/S2	Collette *et al.* 2009

***CCV Fractionation and Immunoblotting***

S-1000 Sephacryl size exclusion chromatography was used to isolate CCVs from SL5838 yeast as described previously (Lemmon *et al.* 1988). Fractions were analyzed using standard SDS-PAGE and immunoblotting techniques. For proteomic analysis from the *chc1∆* strain (SL12), identical S-1000 CCV fractions were pooled and prepared as described previously (Ding, Segarra *et al.* 2016). Chc1p was detected using mouse monoclonal antibodies developed previously (Lemmon *et al.* 1988) and Atg27p was detected using anti-HA monoclonal antibodies (1:5000, Roche Applied Sciences, Catalog Number 11583816001). Atg9p was detected using polyclonal antibodies obtained from the Ohsumi Lab (Yamamoto *et al.* 2012). Immunoblots were developed using an Odyssey Infrared Imaging System (LiCor) using appropriately conjugated secondary antibodies (Rockland).

***Reproducibility***

Figure 1 depicts one representative biological replicate. Overall, two biological replicates were performed each for Atg27p detection in CCVs from auxilin-depleted and WT strains (identification through mass spectrometry or immunoblotting). Two technical replicates were performed for Atg9p immunoblotting in CCV-enriched fractions from the auxilin-depleted strain.
